# Curing Kinetics of Bioderived Furan-Based Epoxy Resins: Study on the Effect of the Epoxy Monomer/Hardener Ratio

**DOI:** 10.3390/polym14235322

**Published:** 2022-12-05

**Authors:** Angela Marotta, Noemi Faggio, Cosimo Brondi

**Affiliations:** 1 Department of Chemical, Materials and Production Engineering, University of Naples Federico II, Piazzale Vincenzo Tecchio, 80, 80125 Naples, Italy; 2 Institute for Polymers, Composites and Biomaterials (IPCB)—CNR, Via Campi Flegrei 34, 80078 Pozzuoli, Italy

**Keywords:** epoxy resins, anhydride curing agent, curing kinetics, calorimetry, bio-based epoxy, furan-based epoxy

## Abstract

The potential of furan-based epoxy thermosets as a greener alternative to diglycidyl ether of Bisphenol A (DGEBA)-based resins has been demonstrated in recent literature. Therefore, a deep investigation of the curing behaviour of these systems may allow their use for industrial applications. In this work, the curing mechanism of 2,5-bis[(oxiran-2-ylmethoxy)methyl]furan (BOMF) with methyl nadic anhydride (MNA) in the presence of 2-methylimidazole as a catalyst is analyzed. In particular, three systems characterized by different epoxy/anhydride molar ratios are investigated. The curing kinetics are studied through differential scanning calorimetry, both in isothermal and non-isothermal modes. The total heat of reaction of the epoxy resin as well as its activation energy are estimated by the non-isothermal measurements, while the fitting of isothermal data with Kamal’s autocatalytic model provides the kinetic parameters. The results are discussed as a function of the resin composition. The global activation energy for the curing process of BOMF/MNA resins is in the range 72–79 kJ/mol, depending on both the model used and the sample composition; higher values are experienced by the system with balanced stoichiometry. By the fitting of the isothermal analysis, it emerged that the order of reaction is not only dependent on the temperature, but also on the composition, even though the values range between 0.31 and 1.24.

## 1. Introduction

Epoxy thermoset resins are polymeric materials widely used in the industrial field thanks to their excellent mechanical and thermal properties, as well as their chemical resistance [1]. Specific applications require remarkable structural characteristics of epoxy resins, which in turn are dictated by the nature of the epoxy monomer and hardener, as well as the network feature developed upon curing [2]. The curing reaction proceeds through different mechanisms depending on the nature of the crosslinking agent and the catalyst, as well as on the curing conditions [2].

In particular, the latter have a significant impact on the network structure, and as a result, the deeper the understanding of the curing mechanism and kinetics of an epoxy resin, the greater the control on the final properties of the material [3]. Several experimental techniques and methods are used to assess chemical reaction kinetics, such as Fourier transform infrared spectroscopy (FTIR) [4,5], nuclear magnetic resonance (NMR) [6,7], and dynamic mechanical analysis [8,9]. In this sense, one of the most widely used techniques to identify the kinetic parameters, such as the rate constant, activation energy, and reaction order, associated with a curing reaction is differential scanning calorimetry (DSC), used [10] under both isothermal and dynamic conditions [11]. The widespread use of the DSC technique is due to its higher feasibility compared to FTIR and NMR. In fact, NMR analysis requires highly expensive and difficult-to-use apparatus. FTIR instead, despite the easiness of use, is a less accurate technique due to the possible overlapping of the characteristic peaks associated with the reacting groups [12]. In fact, Shnawa [13] investigated the structure of a tannin-based epoxy resin (TER) by means of FTIR. Although this equipment provided some insights about the chemical architecture of this compound, it was possible to investigate the formation of the crosslinked network by DSC only. In particular, it was observed that the TER was successfully cured with an amine-based curing agent. Moreover, DSC analysis confirmed that TER blended to commercial epoxy resin could act as a curing accelerator by monitoring the exothermicity of the curing peaks and the completion of the curing temperatures. In another work, Yang and co-authors [14] demonstrated that the addition of a phosphorous/imidazole-containing compound to epoxy resins significantly decreases the curing time.

In recent studies carried out by the authors, the curing behavior of 2,5-bis[(oxiran-2-ylmethoxy)methyl]furan (BOMF) with methyl nadic anhydride (MNA) has been already reported [12]. This resin has been applied as a tinplate coating, showing excellent adhesion and chemical resistance, thus being a good substitute for DGEBA-based products in the field of metal packaging [15]. An interesting aspect of such systems relies on the possibility of tuning their properties by changing the epoxy/anhydride ratio, the nature of the catalyst, or by including an opportunely selected inorganic filler.

Considering the already well-established use of carbohydrate derivatives concerning the category of thermosetting epoxy resins, furanic compounds attracted an increased interest as a potential alternative in the replacement of oil-based ones. Under this perspective, furan-based epoxy resins have been studied in the last years but have not been fully exploited yet. To assess the potential use of these compounds, a study on the reaction kinetics by FTIR [12] and the coating properties [15] was already performed. To our knowledge, a deeper study on the reaction mechanisms and the kinetic parameters of BOMF/MNA has not been performed yet.

Taking all the above considerations into account, the goal of this work is not only to provide insight into the curing kinetics of the biobased BOMF/MNA resin, but also to evaluate the effects of different epoxy/anhydride combinations in the reacting mixture. To this aim, DSC has been selected as a tool to carry out these studies. In particular, non-isothermal and isothermal DSC analyses were conducted, and different thermokinetic methods were used to calculate the characteristic kinetic parameters of the BOMF/MNA curing process as function of the resin composition.

## 2. Materials and Methods

### 2.1. Materials

In this work 2,5-bis[(oxiran-2-ylmethoxy)methyl]furan (BOMF) was used as a biobased epoxy monomer and methyl-5-norbornene-2,3-dicarboxylic anhydride (methyl nadic anhydride, MNA, 90%), purchased from Sigma-Aldrich (Darmstadt, Germany), was used as a curing agent. To ensure the obtainment of a crosslinked material, 2-methylimidazole (2-MI, 99%), purchased from Acros Organics (Geel, Belgium), was used as an initiator. MNA and 2-MI were used as received without further purification, while BOMF was synthesized following the procedure present in the literature [12].

### 2.2. Sample Preparation

Epoxy/anhydride mixtures were prepared by mixing at room temperature the epoxy monomer and the curing agent in the proper amounts and the initiator to the extent of 0.5 wt.% of the total amount of the epoxy monomer and anhydride. The weights of BOMF (the epoxy monomer) and MNA (the anhydride curing agent) were calculated to obtain different molar epoxy-to-anhydride ratios, namely 1/1 (stoichiometric ratio), 1/0.8 (epoxy excess), and 0.8/1 (anhydride ratio).

### 2.3. Differential Scanning Calorimetry

Differential scanning calorimetric analyses (DSC) were performed by means of a DSC Q2000 (TA Instrument, New Castle, DE, USA) equipped with a refrigerator cooling system (RCS). The analyses were performed on samples of about 7–8 mg sealed in aluminum pans immediately after the preparation of the mixtures. The samples were stored at −30 °C for a maximum of 2 days. Measurements under inert nitrogen flux (50 mL/min) in both non-isothermal conditions, heating from 25 to 200 °C at heating rates of 1, 1.5, 3, 5, and 10 °C/min, and isothermal conditions, at temperatures of 90, 100, 110, 120, and 130 °C, were performed.

## 3. Modeling

The degree of cure (α) corresponds to the integrated area of a specific DSC peak and can range from 0 to 1. At the current time (*t*), α can be measured as reported [16,17,18]:

(1) $$\alpha \left(t\right)=\frac{1}{\Delta H_{tot}}\int_{0}^{t}dH=\frac{\Delta H\left(t\right)}{\Delta H_{tot}}$$

where ∆*H*(*t*) = $\int_{0}^{t}dH$ is the reaction enthalpy at the time *t* and ∆*H_tot_* is the total reaction enthalpy [19,20]. As commonly performed in kinetic analyses conducted by DSC [16], it is assumed that there is a direct proportionality among the heat flow (*dQ*/*dt*) detected during the analysis and the conversion rate (*dα*/*dt*) [21] according to the following equation:

(2) $$\frac{dQ}{dt}=Q_{tot}\frac{d\alpha }{dt}$$

In the case of the isothermal mode, the functional dependencies of the conversion rate can be regarded as the product of two basic functions, respectively, related to the dependency on the temperature and the degree of cure:

(3) $$\frac{d\alpha }{dt}=k\left(T\right)\;f\left(\alpha \right)$$

where *k*(*T*) is the rate constant and *f*(*α*) is the reaction model. According to the transition-state theory [22], the activation energy represents the difference in the energy amount between the reactant molecule at its initial configuration and the corresponding transition-state molecule in its activated configuration. In the context of a chemical reaction, this quantity can be formally regarded as the minimum amount of energy required to activate the reactant molecules so that they can undergo chemical transformations [23]. Under this perspective, the activation energy can be enclosed in a rate constant that is dependent on the temperature, not the concentration of the reactants, and can be formally expressed by the Arrhenius equation:

(4) $$k\left(T\right)=A\;e^{\left(\frac{-E_{a}}{RT}\right)}$$

where *A* is a preexponential factor, *R* is the gas constant, *T* is the temperature, and *E_a_* is the activation energy. The reaction model *f*(*α*) reflects the reaction mechanism and, in the specific case of an epoxy curing reaction, it generally takes the form of a *n*th-order kinetic model (Equation (5)) or an autocatalytic process (Kamal’s model, Equation (6)) [23,24,25]:

(5) $$f\left(\alpha \right)={\left(1-\alpha \right)}^{n}$$

(6) $$f\left(\alpha \right)=\alpha^{m}{\left(1-\alpha \right)}^{n}$$

In general, the *n*th-order and the autocatalytic model provide a suitable description of the behavior of the thermosetting materials [26,27]. In particular, the *n*th-order kinetics report a reaction rate proportional to the n exponent of the reactant concentration. In this case, the model assumes that only one reaction occurs during the curing process and, therefore, can lead to some limitations since more simultaneous reactions can take place during the whole process. With reference to the autocatalytic model, reaction products also participate in the reaction, leading to a reaction rate increase during the initial stage, reaching a maximum, and then decreasing [16,28].

The abovementioned models imply that a system achieves a full extent of cure, that is, *α* = 1. As a complete cure is not always observed in epoxy curing systems, Equations (5) and (6) can be manipulated so that the kinetic models are also applicable to incomplete cure, and Equations (7) and (8) are, respectively, obtained:

(7) $$f\left(\alpha \right)={\left(\alpha_{max}-\alpha \right)}^{n}$$

(8) $$f\left(\alpha \right)=\alpha^{m}{\left(\alpha_{max}-\alpha \right)}^{n}$$

where *α_max_* is the maximum degree of conversion (experimentally measured) and must be implemented to cope with the incomplete curing reaction. As these concepts are generally valid in the simple case of one-step chemical reactions, the aforementioned considerations and the formalism introduced for the activation energy can be extended to complex multistep reactions, taking into account the rate-controlling step. In our case, for multistep reaction mechanisms, the reaction rate can be expressed as a function of more than one rate constant. Indeed, Kamal’s model can be rearranged and expressed as reported in Equation (9) [29]:

(9) $$\frac{d\alpha }{dt}=\left(k_{1}+k_{2}\alpha^{m}\right){\left(\alpha_{max}-\alpha \right)}^{n}$$

where *k*_1_ is the rate constant of a catalyzed *n*-order reaction and *k*_2_ is the rate constant of an autocatalytic *m*-order reaction.

When dealing with the non-isothermal mode, the formalism implied for the expression of the conversion rate in the case of the isothermal analysis (Equation (3)) can be adopted in the same way to express the functional dependencies. As heating experiments are performed at constant heating rates *β* = *dT/dt* > 0, the conversion rate can be expressed as the following:

(10) $$\frac{d\alpha }{dt}=\frac{1}{\beta }k\left(T\right)\;f\left(\alpha \right)$$

Typically, the rate of reaction at a constant heating rate as function of temperature exhibits a relative maximum within the temperature interval under study. Moreover, the rate constant can be expressed by the Arrhenius equation also in the case of the non-isothermal analysis. In our case, the Kissinger and the Flynn–Wall–Ozawa models have been applied for the determination of the energy of activation of the overall reaction.

The Kissinger model [30,31] retrieved the *E_a_* at several peak temperatures (*T_p_*) due to the different applied heating rates *β*, assuming that the reaction rate in a constant conversion is solely dependent on the temperature. In this case, the following linear relationship is reported:

(11) $$\ln\left(\frac{\beta }{T_{p}^{2}}\right)=-\frac{E_{a}}{RT_{p}}+ln\left(\frac{AR}{E_{a}}\right)$$

Under these conditions, Kissinger [30,31,32] reported that, for a series of non-isothermal tests, the following dependency of the heating rate on the inverse of the peak temperature holds true:

(12) $$\frac{d\;\ln\left(\beta /T_{p}^{2}\right)}{d\left(1/T_{p}\right)}=-\frac{E_{a}}{R}$$

In the case of the Flynn–Wall–Ozawa model, the Arrhenius rate law was integrated and then the Doyle approximation [33,34] was applied to obtain Equation (13). According to ASTM E698, [35] also in this case, the analysis was performed on several heating rates, with *T_p_* being independent of the heating rate:

(13) $$\ln\left(\beta \right)=-1.052\;\frac{E_{a}}{RT_{p}}-const.+ln\left(\frac{A\;E_{a}}{R\;f\left(\alpha \right)}\right)$$

where *const.* stands for a constant term depending on the integration method applied to the Arrhenius law, while *f*(*α*) is the reaction model. As well in this case, for a series of non-isothermal tests, the following dependency of the heating rate on the inverse of the peak temperature holds true: [36,37]

(14) $$\frac{d\;\ln\left(\beta \right)}{d\left(1/T_{p}\right)}=-1.052\;\frac{\;E_{a}}{R}$$

Both Equations (12) and (14) suggest two alternative ways to estimate the activation energy. Through the linear fitting of data derived by dynamic DSC measurements, from a plot of ln(*β*/$T_{p}^{2}$) [or ln(*β*)] versus 1/*T_p_*, it is possible to determine the apparent activation energy value with further insight on the preexponential factor *A* in the case of the Kissinger method.

## 4. Results

**Figure 1:**
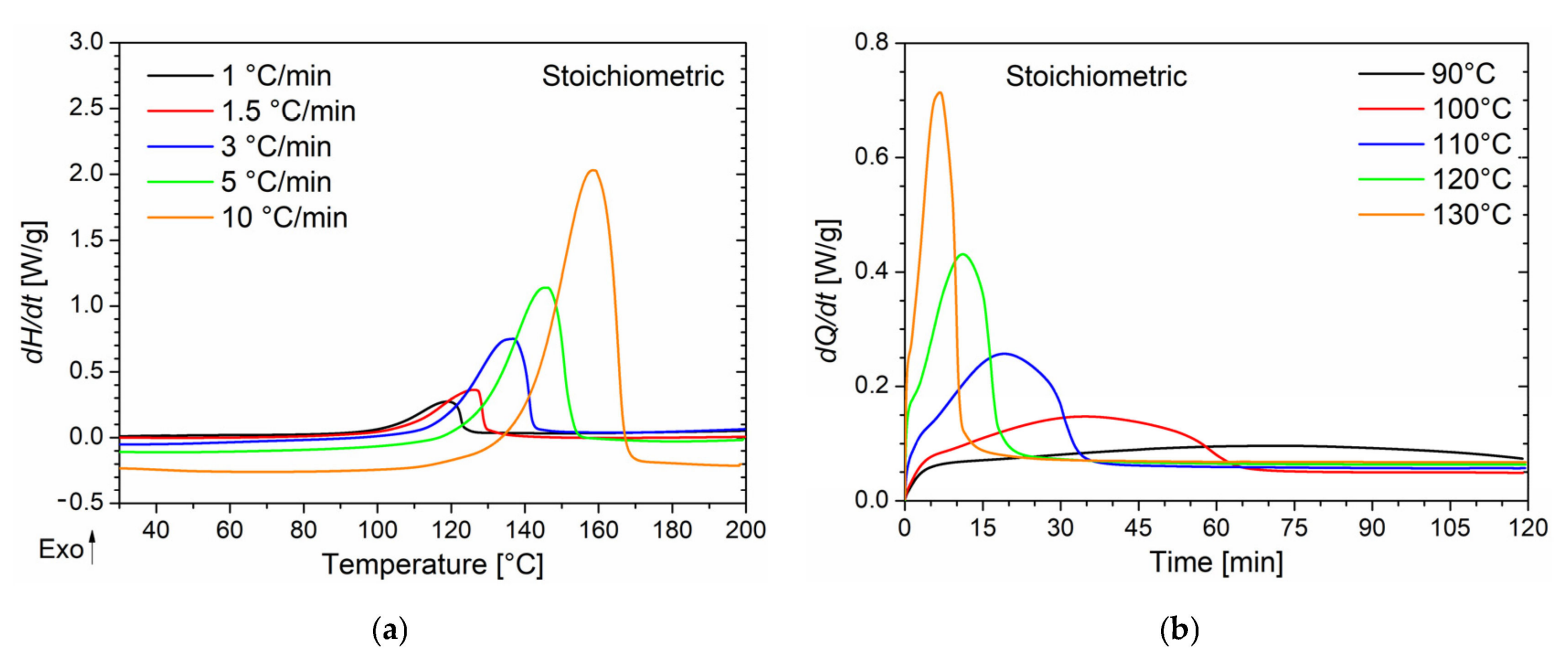

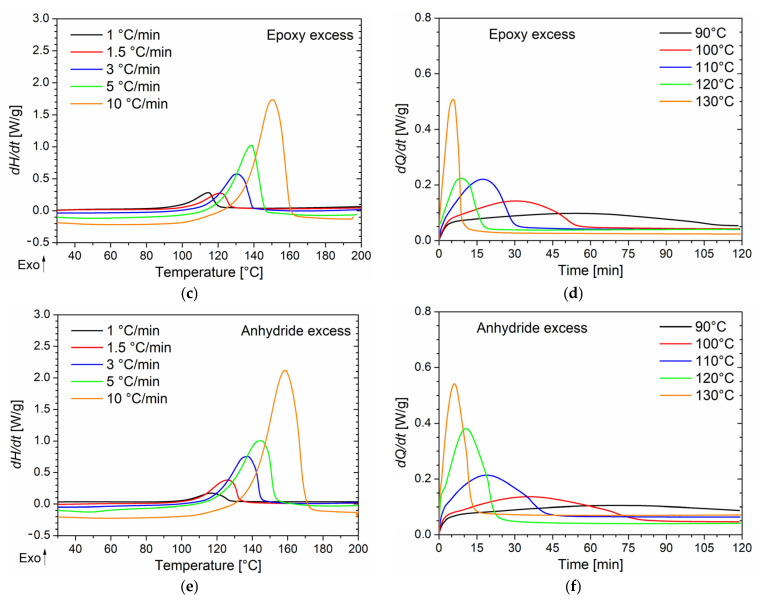
Non-isothermal DSC thermograms of BOMF/MNA resins with (**a**) a stoichiometric epoxy/anhydride ratio, (**c**) epoxy excess, and (**e**) anhydride excess. Dynamic DSC analyses were conducted at five heating rates (i.e., 1 °C/min, 1.5 °C/min, 3 °C/min, 5 °C/min, and 10 °C/min) for each sample. Isothermal DSC thermograms of BOMF/MNA resins with (**b**) a stoichiometric epoxy/anhydride ratio, (**d**) epoxy excess, and (**f**) anhydride excess at five different temperatures (i.e., 90, 100, 110, 120, and 130 °C) for each sample.

Differential scanning calorimetry was used to investigate the curing behavior of epoxy/anhydride mixtures with different molar ratios in both dynamic and isothermal conditions. DSC thermograms collected at different heating rates (from 1 to 10 °C/min) for the three formulations (stoichiometric, epoxy excess, and anhydride excess) are respectively reported in Figure 1a,c,e with their respective peak temperature (*T_p_*) values listed in Table 1. The absence of any residual post-cure reactions was certified by the absence of a further exotherm peak during the second heating ramp performed on all the studied samples. As no further peak was detected, samples were assumed as fully cured during the dynamic run. Therefore, the total heat of reaction Δ*H_tot_* was evaluated using the DSC analysis conducted at 10 °C/min for each sample. The calculated values of Δ*H_tot_* are 353.7, 301.1, and 377.2 J/g for the samples at a stoichiometric epoxy/anhydride ratio, epoxy excess, and anhydride excess, respectively. The thermal stability of the same three epoxy/anhydride systems at different molar ratios was investigated by means of thermogravimetric analysis (TGA) and reported in the supplementary information of a previous work [12]. The systems did not experience any thermal degradation in the range of temperatures utilized in the DSC analyses.

All the systems exhibit a single exothermic peak with a *T_p_*, which shifts to higher temperatures by increasing the heating rate regardless of the epoxy/anhydride ratio. Furthermore, when an epoxy excess is used, at each heating rate, *T_p_* is about 5 °C lower than that of other samples. It has been reported that a lower *T_p_* appears to be an indicator of higher curing reactivity [38]; however, this occurrence will be confuted by considerations regarding the activation energy of the curing reaction, reported further in this section. This trend can be, instead, explained by the increased mobility of the growing macromolecular units associated with the decrease in the mixture viscosity when the epoxy monomer is added in stoichiometric excess [12].

**Figure 2:**
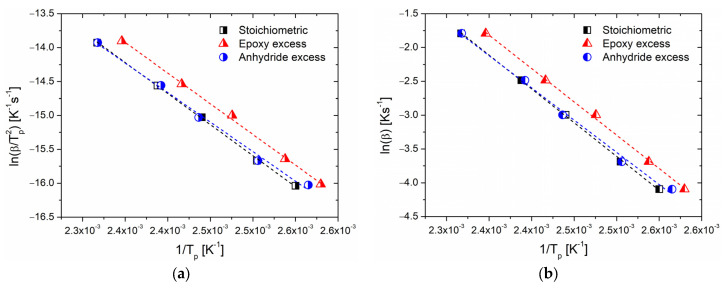
Plots of the heating rate versus 1/*T_p_*. Dash lines represent the linear fit according to (**a**) Kissinger (Equation (11)) and (**b**) Ozawa (Equation (13)).

As both the Kissinger and Ozawa approaches are based on fitting procedures applied at various heating rates [30,31,32,35,36,37] and rely on the assumption that the extent of reaction *α* at the peak temperature (*T_p_*) is constant and independent of such heating rates, these methods were applied to calculate the activation energy as solely a function of the epoxy/anhydride ratio and provide a better elucidation on the stoichiometry effects. Figure 2a reports the values of ln(*β*/$T_{p}^{2}$) as function of 1/*T_p_*; these data were linearly fitted, and the obtained parameters were used to evaluate the activation energy as well as the preexponential factor *A* for the overall reaction, according to Equation (11). Similarly, Figure 2b reports the linear plot of ln(*β*) versus 1/*T_p_* according to the Ozawa model (Equation (13)), and its linear fitting can be observed. The calculated values of the kinetic parameters are summarized in Table 2.

Both the Kissinger and Ozawa models return similar values of activation energy for systems with a stoichiometric ratio and an excess of epoxide, indicating the same global reactivity of the two systems. In particular, it can be observed that the lower peak temperatures when epoxides are added in larger quantities, compared to those of mixtures at a stoichiometric ratio, may suggest an enhanced reactivity of the samples at epoxy excess. However, we remark that this observation may lead to an erroneous evaluation of the tendency of reaction of the system. On the other hand, for the system with an excess of anhydride, the *E_a_* is lower, and this occurrence indicates an inhibited reactivity of the system despite the fact that *T_p_* is comparable with the stoichiometric system. The *E_a_* values evaluated with the Kissinger method are systematically lower than those evaluated with the Ozawa method, as expected by the comparison with similar data reported in the literature [30,31,32,35,36,37,39,40,41].

In the case of the isothermal DSC tests conducted on the BOMF/MNA resins at different epoxy/anhydride ratios, to prevent vitrification as much as possible, five temperature values lower than the onset temperature value of the cure peak detected in the dynamic DSC analyses were chosen. In Figure 1b,d,f, the thermograms of the isothermal analyses are reported in which the heat flows as a function of time (directly related to the rate of conversion according to Equation (2)) are depicted. As can be seen, when decreasing the curing temperature, a longer reaction time is needed for the system to complete the cure and to reach the maximum conversion rate. This behavior is characteristic of autocatalytic reaction kinetics [18,42]. As already observed, reactions characterized by n-order kinetics show a peak in isothermal scans, eventually approaching zero for longer times regardless of the temperature. In our case, all systems cured at 90 °C did not fully react during the analysis time (two hours). It was proved by performing a heating scan on the samples that underwent the isothermal analysis in which a residual cure was detected. In addition, lower peak temperatures and higher values of the conversion rate are associated with an increase in the curing temperature. For all the samples cured above 90 °C, no post-cure reactions were detected by dynamic scans performed on the samples that underwent isothermal tests.

**Figure 3:**
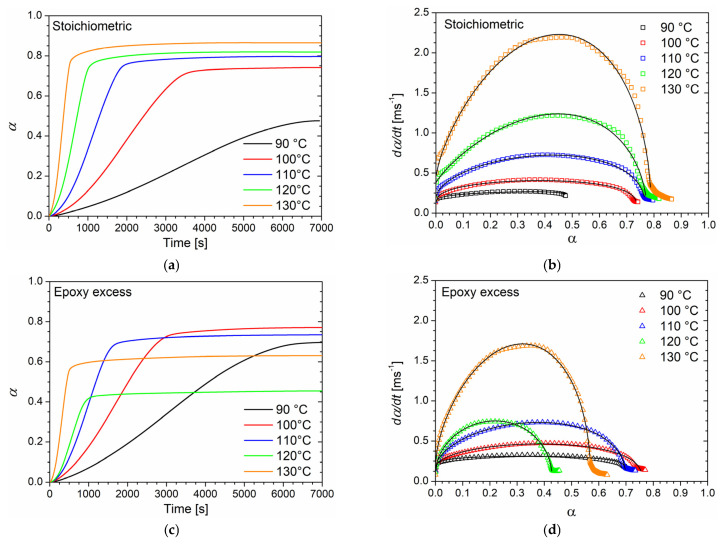

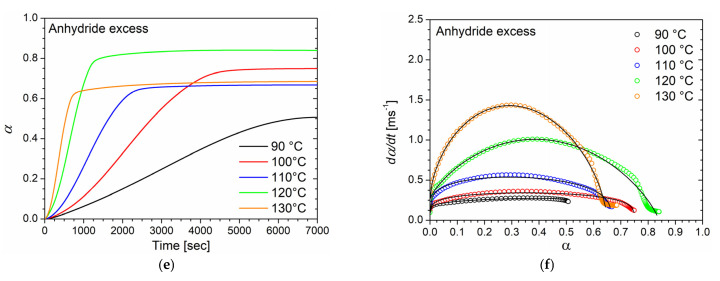
Plots of the degree of conversion (*α*) as a function of time and conversion rate (*dα*/*dt*) as function of conversion for epoxy/anhydride mixtures with (**a**,**b**) a stoichiometric ratio, (**c**,**d**) epoxy excess, and (**e,f**) anhydride excess, respectively. Solid lines in (**b**,**d**,**f**) represent the fitting curves of the experimental data by the Kamal model (Equation (9)).

The kinetic parameters of the curing reaction were calculated using the analyses of thermograms obtained from the isothermal DSC experiments. To this regard, an accurate baseline construction was required to obtain consistent results relevant to the kinetic analysis. In this work, for all the samples, a baseline was constructed, according to the procedure reported by Barton et al. [10], by performing an isothermal scan on the post-cured samples. The degree of cure as a function of time for each temperature (Figure 3a,c,e) was determined relating the reaction enthalpy obtained by isothermal analysis to the Δ*H_tot_* value derived from the dynamic measurements previously reported, following Equation (1).

The sigmoidal shape of the α–time curves, especially for low temperatures, is characteristic of autocatalytic processes for which the reaction rate is relatively low at the beginning of the process, and the conversion degree only slowly increase due to the initial reaction steps. As new functional groups, which can catalyze the curing process, are created by the first reaction steps, the cure degree rapidly increases until a high degree of cure is achieved. At this time, the reaction rate drastically decreases, and α reaches a plateau. Nevertheless, no sample reaches the maximum degree of cure (Table 3). In particular, a linear increase in *α_max_* with temperature can be noticed for the system with an equimolar epoxy/anhydride ratio, while systems with unbalanced stoichiometry exhibited a non-linear behavior.

Reaction rate data were fitted to the Kamal model (Equation (9)) by minimizing the least square error function to evaluate the *k*_1_, *k*_2_, *m*, and *n* kinetic parameters with no prior assumption for the total reaction order *m* + *n*. The best-fitting values obtained for the kinetic parameters at various curing temperatures are listed in Table 4. In Figure 3b,d,f the experimental data (symbols) are compared to the fitted data (black solid lines). Overall, the curing degree rate is adequately described by autocatalytic behavior.

The total reaction order *m* + *n* as well as the rate constants show an increasing trend with the curing temperature at each epoxy/anhydride ratio.

**Figure 4:**
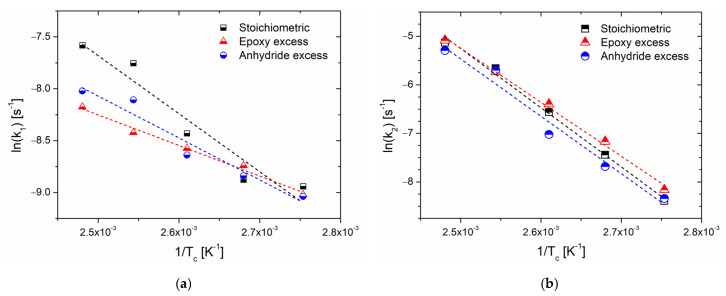
Linear plots of the logarithm of the rate constants, (**a**) ln(*k*_1_) and (**b**) ln(*k*_2_)), obtained by fitting the isothermal data with the Kamal model (Equation (9)) as function of the inverse of the cure temperature (1/*T_c_*) at different epoxy/anhydride ratios.

Further analysis can be carried out on the obtained values for the rate constants *k*_1_ and *k*_2_, which express, respectively, the influence of the initiation step on the overall reaction and the dependence of the reaction rate on the species formed during the reaction steps (that also act as catalysts in autocatalytic processes) [18,42]. As the rate constants comply with the Arrhenius law (Equation (4)), by plotting their logarithm against the inverse of the cure temperatures (Figure 4), it is possible to gather values of the preexponential factor *A* and the activation energy *E_a_* for the single-reaction processes (Table 5).

## 5. Discussion

One of the key factors influencing the morphological changes in the physical structure during thermosetting processes is the curing kinetics. In particular, the epoxy/anhydride crosslinking mechanism is extremely complex, as it involves the simultaneous occurrence of competitive reactions, such as etherification and esterification reactions, which globally lead to the network formation [39,43,44].

**Scheme 1:**
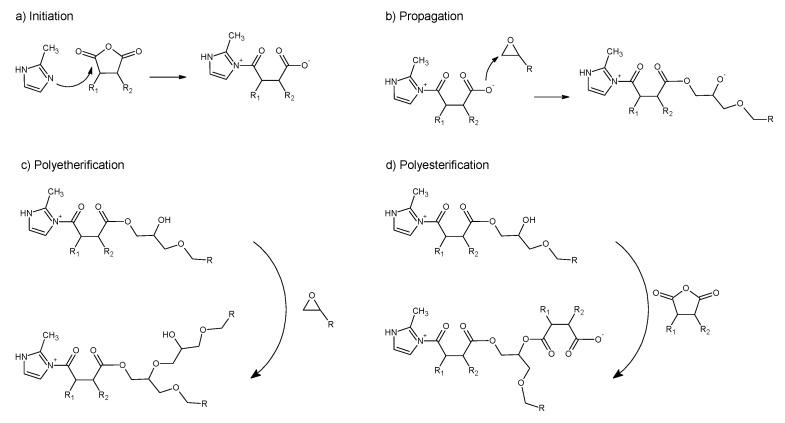
Reaction steps of epoxy/anhydride cure: (**a**) initiation, (**b**) propagation, (**c**) polyetherification and (**d**) polyesterification.

When anhydrides are used as curing agents in presence of imidazole as an initiator, the first reaction step is the initiation, a nucleophilic attack of the imidazole nitrogen to the anhydride group (Scheme 1a) that generates a zwitterion intermediate containing a quaternary nitrogen cation and an active oxyanion. The latter reacts with the anhydride or the epoxy group, giving rise to a carboxylate anion (Scheme 1b), which initiates chain-wise polymerization [43,44,45]. The formed anion can further react both with an epoxy group (polyetherification, Scheme 1c) or with another anhydride (polyesterification, Scheme 1d), propagating the chain growth through an alternating copolymerization anionic mechanism.

Due to the complexity of the mechanism underlying the generation of the thermosetting network of epoxy/anhydride resins, a single-step reaction mechanism is unlikely to correctly fit the experimental data. Moreover, the continuous generation of reactive groups throughout the propagation steps suggests the autocatalytic behavior of the epoxy/anhydride reaction. In fact, the autocatalytic nature of the BOMF/MNA curing reaction clearly is suggested by the shape of the thermograms derived from isothermal DSC analysis: the presence of a maximum at different times, depending on the cure temperature, is particular to autocatalytic processes [46,47,48].

Non-isothermal DSC analyses were elaborated by means of two different methods to derive information about the activation energy of the overall curing process. As expected, the Kissinger and Ozawa methods did not return the same value of *E_a_*, [40,41] but the latter are reasonable values for an epoxy/anhydride cure, in trend with the reported literature [39,49]. In the specific case of BOMF/MNA, if an excess of anhydride is used, even though the maximum reaction rate is reached at a temperature comparable with the systems with a stoichiometric epoxy/anhydride molar ratio, the system is more reactive (lower activation energy, Table 2). An accelerating effect is indicated by a decrease in the activation energy since less energy from the reacting components is required to complete the reaction [40]. This finding could be related to the higher number of initiation sites formed in the presence of higher amounts of anhydride [12]. Consequently, the number of propagating chains is increased, and so is the number of reactive sites, with an autocatalytic effect responsible for a globally faster reaction (reflected also by the lower value of *A^k^*, Table 2). We remark that the activation energy only provides data on the reaction rate, not about the reaction mechanism, and that both the Kissinger and Ozawa methods provide a single value of the activation energy for the whole process despite the fact that, in complex systems such as epoxy/anhydride resins, this parameter fluctuates with the curing period.

The isothermal analysis of the curing process can, instead, give a major degree of detail about the reaction mechanisms. Isothermal DSC data were fitted to an autocatalytic model developed by Kamal, which well characterizes systems wherein the reaction rate increases due to the catalytic impact of the groups formed during the reaction itself, to define the relationship among conversion, time, and temperature. First of all, the conversion *α* as a function of time was evaluated at different temperatures for the three studied systems (Figure 3a,c,e). The lack of a linear dependance of *α* with temperature for the systems with epoxy excess and anhydride excess, together with the partial extent of cure experienced by all the samples, can be explained by the occurrence, to different extents, of vitrification. The reactivity of the molecules dominates the rate of reaction at the beginning of a curing reaction. As the reaction proceeds, the degree of cure increases and the system first undergoes gelation (i.e., the development of molecular branching), which is related to an increase in viscosity and a subsequent worsening of the molecular mobility. When diffusion-controlled processes overstep the mobility of the reacting groups, the reaction becomes diffusion-controlled and vitrification occurs [50]. By correlating this phenomenon to the conversion degree, we can observe that at low conversion values, the network is far from being formed and the reacting species, still present in high concentrations, can easily diffuse, and the reaction proceeds fast. Once high conversions are reached, long chains are formed, resulting in a viscosity increase which limits the chain mobility; at this point, the reaction becomes diffusion-controlled, and a plateau value for the conversion is reached. When the system vitrifies, increasing the temperature above the glass transition temperature (*T_g_*), the viscosity decreases, and subsequently, the chain mobility is somehow improved, enabling the reaction of the remaining unreacted functional groups [51]. For the systems studied in this work, all the temperatures selected for the isothermal analysis are above *T_g_* [12]. However, due to the narrowness of the cure peak (Figure 1a,c,e), even though the temperature of isothermal analysis is higher than *T_g_*, this latter is still too close to the peak temperature, so vitrification is unavoidable. It is noteworthy to highlight the behavior of the system with an epoxy excess, for which strong vitrification is verified at temperatures of 120 and 130 °C. Using the non-isothermal DSC analysis, it emerged that when BOMF is present in stoichiometric excess, the curing reaction begins at a lower temperature (*T_onset_*) compared to the other systems. In particular, when this system was heated at 10 °C/min to the temperature of 120 °C, the degree of conversion reached was higher than the α experienced by the other samples at the same temperature. This behavior was also reflected in the dependence of conversion on temperature resulting from the isothermal analysis.

The fitting of experimental data with the mechanistic Kamal method was also carried out. As can be seen from Figure 3b,d,f, the model fits well the experimental data, especially for the system with epoxy excess, regardless of the conversion degree. For both mixtures at a stoichiometric ratio and anhydride excess, instead, the fit is in good agreement with the model, particularly at low α values, and a lack of accuracy at higher conversions is noted, when the reaction is controlled by diffusion phenomena rather than by kinetic parameters [52].

The parameters obtained by the fitting of the experimental data, reported in Table 4, reflect, as far as *k*_1_ and *m* are concerned, the contribution of non-autocatalytic phenomena (in this specific case of study, the initiation step). On the other hand, *k*_2_ and *n* account for the contribution of autocatalytic phenomena (i.e., propagation in epoxy/anhydride cure). Regardless of the resin composition, all four parameters generally increase with temperature, even though with a different trend, and *k*_2_ and *m* are systematically higher than *k*_1_ and *n*. Specifically, when the stoichiometry is unbalanced, the order of reaction increases faster with temperature. For the stoichiometric system, instead, *m* increases much more than *n* with temperature, indicating a stronger temperature dependance of propagation compared to initiation. Noteworthy are the values of *m* and *n* for the system with an excess of anhydride: they are almost the same, with an exception made at 90 °C. This occurrence indicates that initiation and propagation proceed simultaneously. Generally speaking, the overall reaction order, *m* + *n*, is in the range of 0.35–1.23 regardless the composition.

Further analysis was conducted on the rate constants obtained by the fitting procedure, extrapolating the values of the activation energy (*E*_*a*1_ and *E*_*a*2_) and the preexponential factor (*A*_1_ and *A*_2_) for *k*_1_ and *k*_2_, respectively. In particular, while the trend of *k*_2_ with temperature is well fitted by a linear trend, the behavior of *k*_1_ is less prominent of a linear behavior, although it is still well described by a linear fit. This is due to the fact that *k*_1_ is only calculated using two or three of the first experimental datapoints, which might result in inaccurate estimated values [52]. As a general consideration, it emerges that the propagation step is much more kinetically disfavored.

All the considerations expressed here can be summarized into practical advice for the application of BOMF/MNA resins. As the curing degree of samples with a stoichiometric ratio among the reactants has an increasing trend with the curing temperature, this composition should be used for applications in which significant differences in curing temperatures might be experienced by different samples, or also at different points of the same sample (i.e., thick samples). If lower curing temperatures must be used, instead, a composition with an epoxy excess should be chosen, which ensures a higher *α_max_* at 90 °C. However, for the cure of this sample, it is mandatory to control the curing temperature, as an uncontrolled increase in the temperature will have a detrimental effect on the degree of cure. On the other hand, when energy consumption is a limiting factor, the use of anhydride excess may be preferred due to the lower activation energy overall required for the curing process.

## 6. Conclusions

The cure kinetics of bioderived furan-based epoxy resins reacted with anhydride in the presence of 2-methylimidazole as an initiator were investigated by both non-isothermal and isothermal differential scanning calorimetry (DSC). The cure kinetics of BOMF/MNA resulted in being dependent on the epoxy/anhydride molar ratio. The main findings of this study can be summed up as follows:

When anhydride is in a molar excess, the promotion of the initiation step leads to an overall improved reactivity. In fact, it was observed that the activation energy of the curing process decreased from 76.2 to 72.4 kJ/mol (Kissinger method) and from 78.9 kJ/mol to 75.3 kJ/mol (Ozawa method) for stoichiometric and anhydride excess, respectively;

The high reactivity of the system, independent of the resin composition, leads to incomplete cure due to vitrification. This occurrence was observed even at the higher curing temperature (130 °C) from the maximum degree of conversion (0.79, 0.57, and 0.65 for stoichiometric, epoxy excess, and anhydride excess, respectively);

Both propagation and initiation are favored by increasing the temperature, but with a different dependence, as a function of the composition.

The autocatalytic Kamal model fits well the experimental data but did not succeed in fully describing the complex epoxy anhydride curing process. The global order of reaction (*m* + *n*) was in the range 0.35–1.24, 0.31–1.11, and 0.33–1.23 for stoichiometric, epoxy excess, and anhydride excess, respectively.

Overall, the observed results enlighten a promising use of these compounds, and a deeper understanding of the reaction kinetics and mechanisms of these systems could help in promoting the application of these biobased materials at an industrial level, addressing the global dependence on oil-derived polymers.

## Figures and Tables

**Table 1:** Peak temperature (*T_p_*) values for the non-isothermal DSC analyses at different heating rates for mixtures with balanced and unbalanced epoxy/anhydride ratios.

	10 °C/min	5 °C/min	3 °C/min	1.5 °C/min	1 °C/min
**Stoichiometric**	158.4 °C	145.6 °C	136.7 °C	126.1 °C	119.0 °C
**Epoxy Excess**	153.1 °C	140.7 °C	130.8 °C	120.9 °C	114.5 °C
**Anhydride Excess**	158.3 °C	144.9 °C	137.3 °C	125.9 °C	116.7 °C

**Table 2:** Values of the global activation energy (*E_a_^K^*) and the preexponential factor (*A^K^*) determined by the Kissinger method and the global activation energy (*E_a_^O^*) determined by the Ozawa method.

	Kissinger		Ozawa
	*E_a_^K^* [kJ/mol]	*A^K^* [s^−1^]	*E_a_^O^* [kJ/mol]
**Stoichiometric**	76.2	1.37 × 10^7^	78.9
**Epoxy Excess**	75.2	1.40 × 10^7^	77.9
**Anhydride Excess**	72.4	4.51 × 10^6^	75.3

**Table 3:** Values of the maximum degree of cure *α_max_* reached after 2 h of isothermal DSC analysis for samples with different epoxy/anhydride ratios.

	*α* *_max_*				
	90 °C	100 °C	110 °C	120 °C	130 °C
**Stoichiometric**	0.48	0.74	0.77	0.78	0.79
**Epoxy Excess**	0.70	0.76	0.70	0.43	0.57
**Anhydride Excess**	0.51	0.75	0.67	0.84	0.65

**Table 4:** Kinetic parameters obtained by least square fitting of the curing degree rate *dα/dt* versus *α* by the Kamal method for systems with different epoxy/anhydride ratios as function of curing temperature (*T_c_*).

	*T_c_* [°C]	*k*_1_ [s^−1^]	*k*_2_ [s^−1^]	*m*	*n*	*m + n*
**Stoichiometric**	90	1.31 × 10^−4^	2.27 × 10^−4^	0.29	0.06	0.35
	100	1.40 × 10^−4^	5.87 × 10^−4^	0.40	0.28	0.68
	110	2.18 × 10^−4^	1.41 × 10^−3^	0.58	0.37	0.95
	120	4.29 × 10^−4^	3.49 × 10^−3^	0.86	0.50	1.36
	130	5.09 × 10^−4^	5.92 × 10^−3^	0.75	0.49	1.24
**Epoxy Excess**	90	1.21 × 10^−4^	2.86 × 10^−4^	0.18	0.13	0.31
	100	1.60 × 10^−4^	7.72 × 10^−4^	0.51	0.36	0.87
	110	1.89 × 10^−4^	1.67 × 10^−3^	0.57	0.40	0.97
	120	2.21 × 10^−4^	3.23 × 10^−3^	0.56	0.47	1.03
	130	2.82 × 10^−4^	6.18 × 10^−3^	0.65	0.46	1.11
**Anhydride Excess**	90	1.19 × 10^−4^	2.39 × 10^−4^	0.28	0.05	0.33
	100	1.45 × 10^−4^	3.76 × 10^−4^	0.27	0.25	0.52
	110	1.77 × 10^−4^	7.49 × 10^−4^	0.27	0.31	0.58
	120	3.02 × 10^−4^	3.35 × 10^−4^	0.79	0.78	1.57
	130	3.28 × 10^−4^	5.03 × 10^−4^	0.59	0.64	1.23

**Table 5:** Values of the preexponential factor (*A*_1_) and activation energy (*E*_*a*1_) from the linear fitting of the rate constant *k*_1_ and values of the preexponential factor (*A*_2_) and activation energy (*E*_*a*2_) from the linear fitting of the rate constant *k*_2_ for systems at different epoxy/anhydride ratios.

	*A*_1_ [s^−1^]	*E*_*a*1_ [kJ/mol]	*A*_2_ [s^−1^]	*E*_*a*2_ [kJ/mol]
**Stoichiometric**	5.49 × 10^2^	46.5	8.83 × 10^10^	101.3
**Epoxy excess**	4.13 × 10^−1^	24.5	6.22 × 10^9^	92.4
**Anhydride excess**	7.64	33.6	2.85 × 10^10^	98.2

## Data Availability

The data presented in this study are available on request from the corresponding author.
